# Supplementary figures and images for: Transport stress induces paradoxical increases in airway inflammatory responses in beef stocker cattle
Source: PLoS One. 2026 Feb 6;21(2):e0328428. doi: 10.1371/journal.pone.0328428 (PMC12880702; doi:10.1371/journal.pone.0328428)

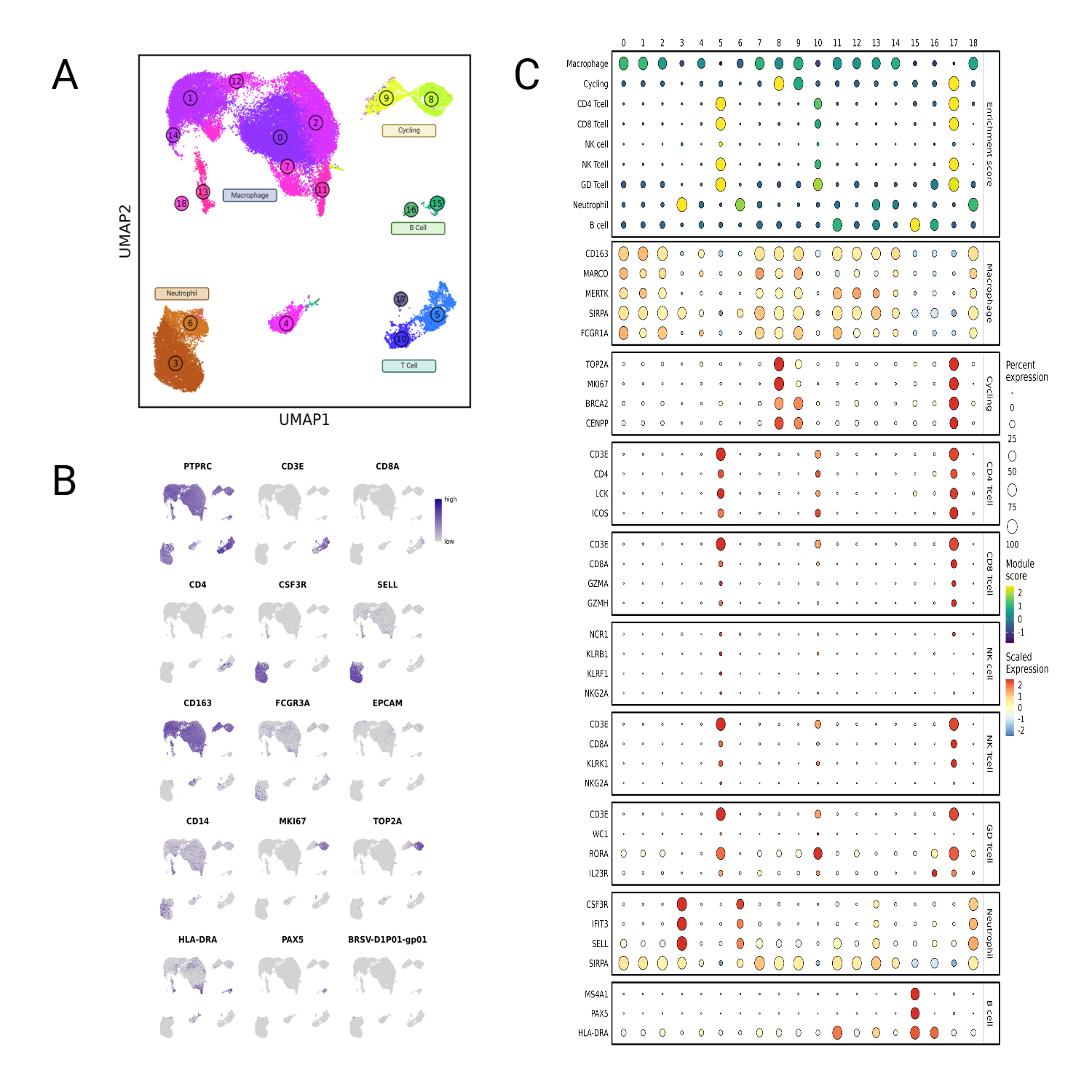

Supplement: S1 Fig — (TIF) [file pone.0328428.s001.tif]

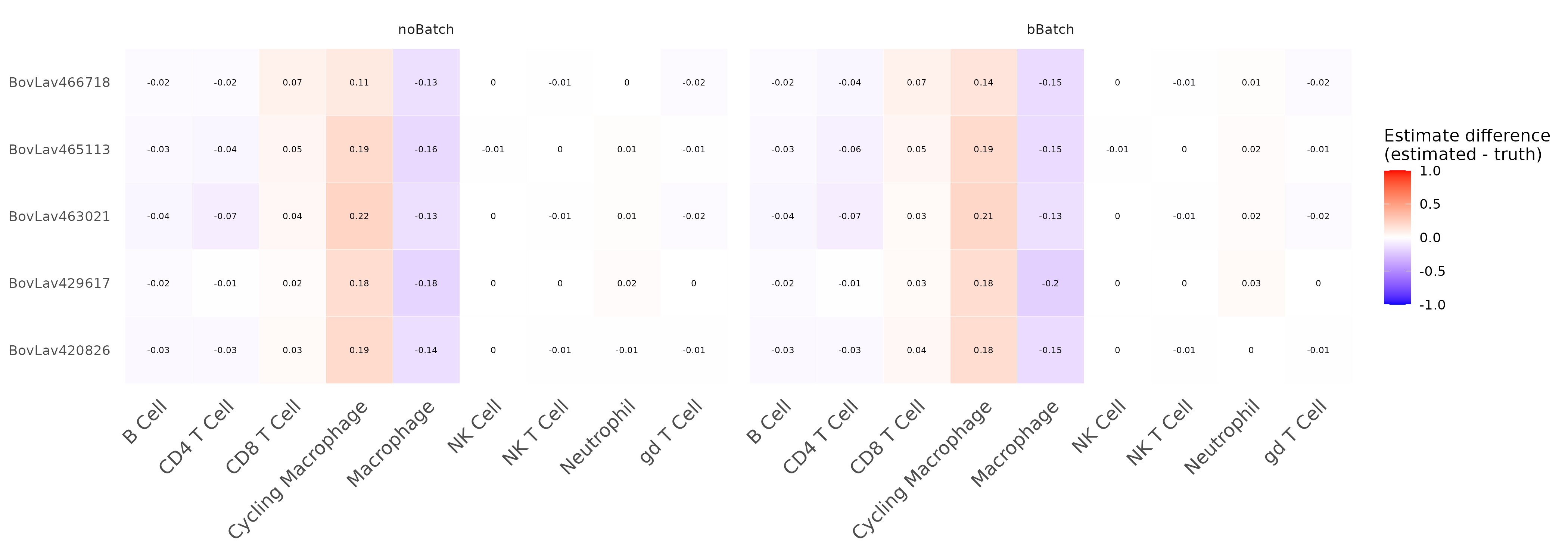

Supplement: S2 Fig — (TIF) [file pone.0328428.s002.tif]

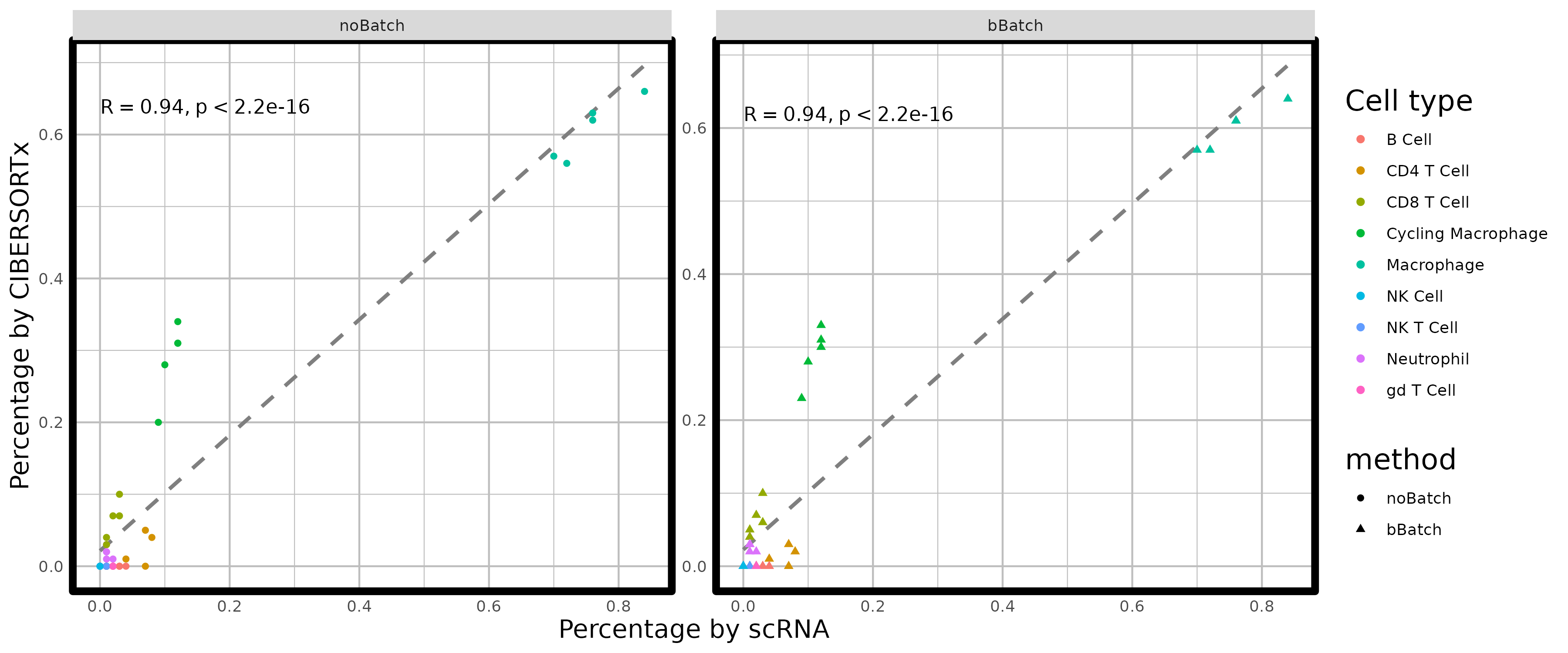

Supplement: S3 Fig — (TIF) [file pone.0328428.s003.tif]

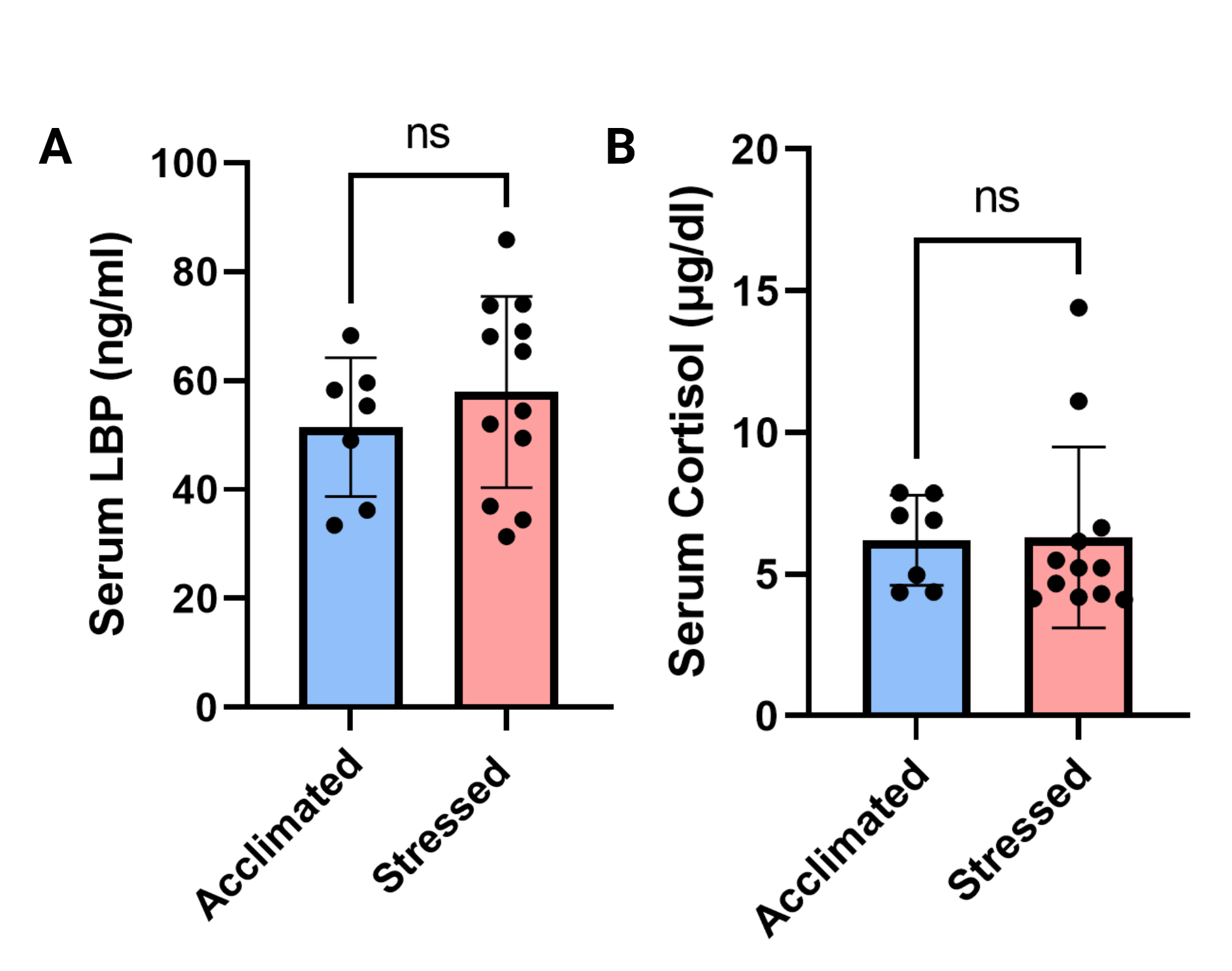

Supplement: S4 Fig — (TIF) [file pone.0328428.s004.tif]

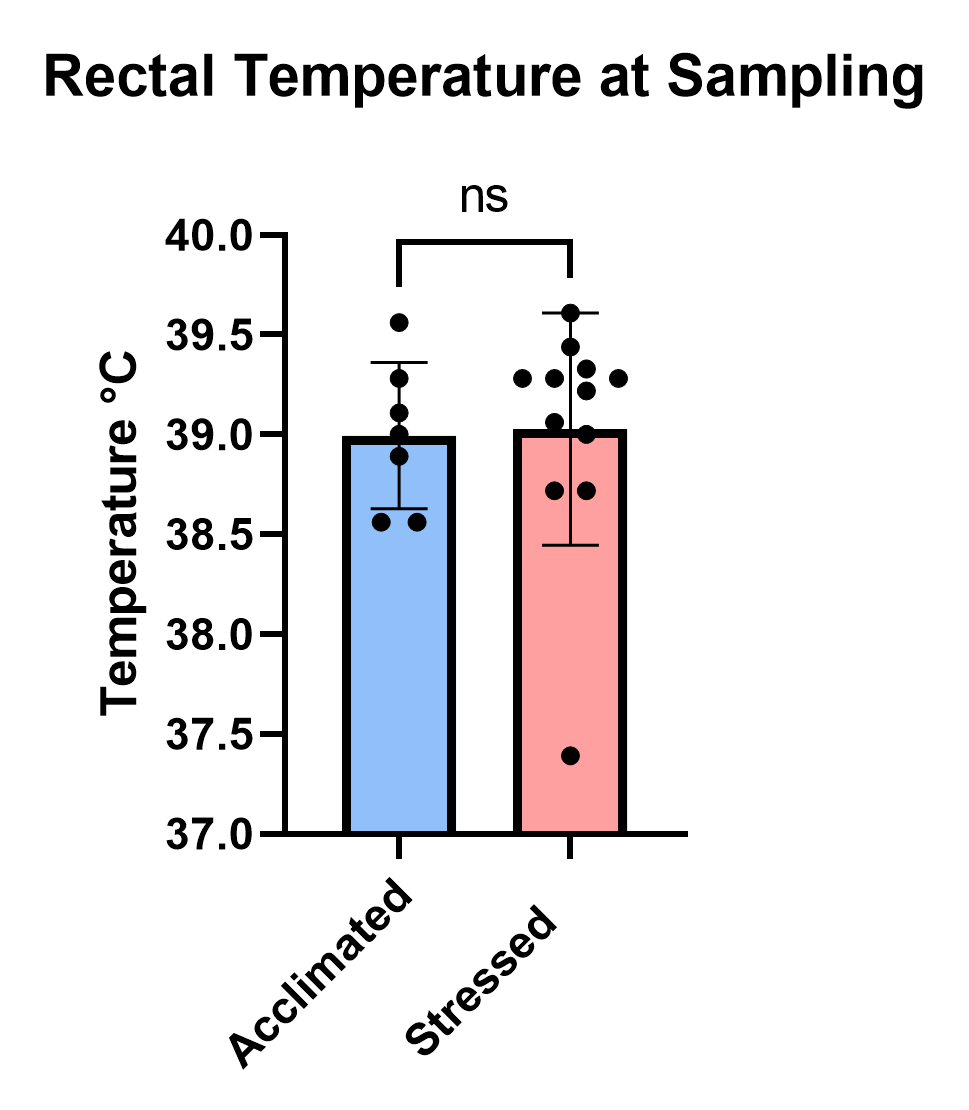

Supplement: S5 Fig — (TIF) [file pone.0328428.s005.tif]

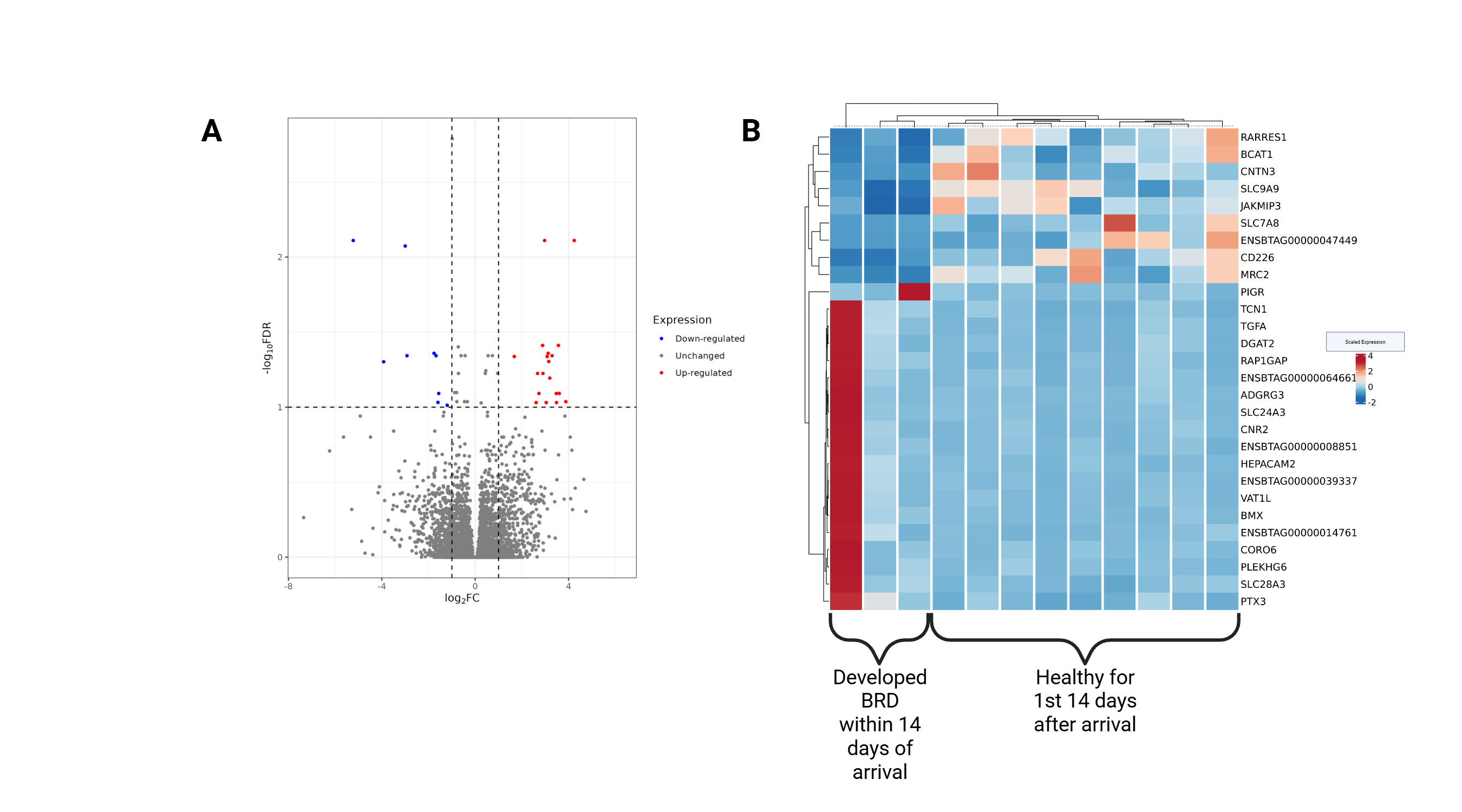

Supplement: S6 Fig — (TIF) [file pone.0328428.s006.tif]

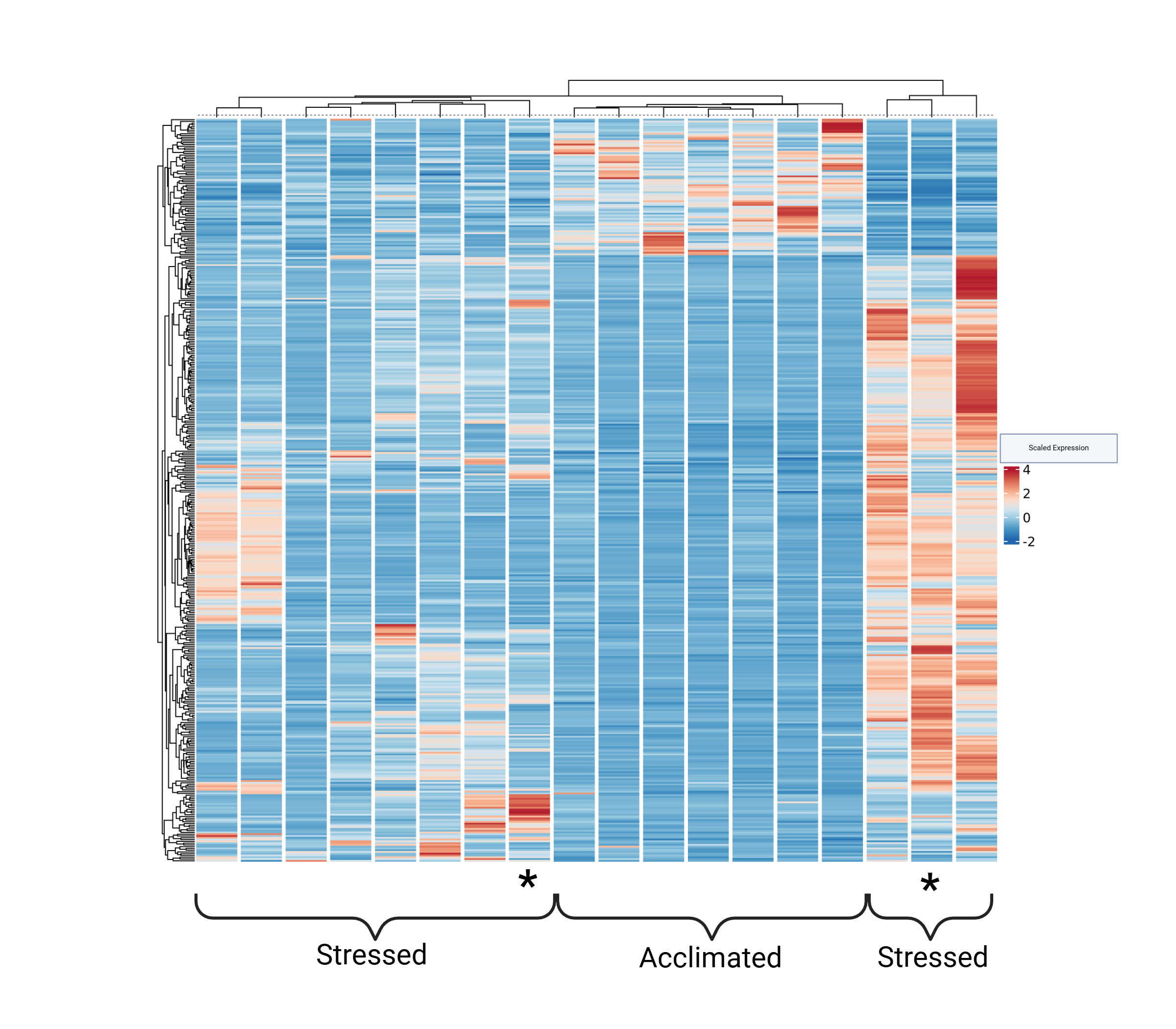

Supplement: S7 Fig — (TIF) [file pone.0328428.s007.tif]
