# Supplementary material for: Transport stress induces paradoxical increases in airway inflammatory responses in beef stocker cattle
Source: PLoS One. 2026 Feb 6;21(2):e0328428. doi: 10.1371/journal.pone.0328428 (PMC12880702; doi:10.1371/journal.pone.0328428)
Supplement: Attachment — Submitted filename: Response to Reviewers.docx [file pone.0328428.s012.docx]

Dear Dr. Abuelo,

Thank you for your consideration of our manuscript entitled, “Transport stress induced paradoxical increases in airway inflammatory responses in beef stocker cattle.” We are very appreciative of the feedback we received from you and both reviewers. We have organized our rebuttal letter into sections: Response to Editorial Comments, Response to Reviewer 1, and Response to Reviewer 2. We have cited where each change has been made based upon their line numbers in the marked-up copy, and in parentheses where they can be found in the unmarked version, (e.g. “change was made on line 85-100 (67-82)”). Please note that in addressing some of the reviewer comments pertaining to the CIBERSORTx methodology and increased metadata request, we have added a significant number of supplemental tables and figures. Because of this, the numbering of many of our tables and figures has changed, as will be reflected in the revised manuscript. Per comments from both Reviewers 1 and 2, we have elected to remove primary Figure 7 from the manuscript, as both reviewers did not feel that it merited inclusion.

We also wanted to acknowledge that we did not originally report the required detail in methodology for the CIBERSORTx predictions included in this study. This was an unintentional oversight, and we have made every effort to document both the markers and identity assignment used in the original single-cell dataset, as well as the correlation of predicted cellular populations with single-cell population proportions. If the Editor or Reviewers have additional requests for CIBERSORTx data support, we will readily provide those as indicated.

**Response to Editorial Comments:**

*1. Please ensure that your manuscript meets PLOS ONE's style requirements, including those for file naming. The PLOS ONE style templates can be found at*

*https://journals.plos.org/plosone/s/file?id=wjVg/PLOSOne_formatting_sample_main_body.pdf and*

[*https://journals.plos.org/plosone/s/file?id=ba62/PLOSOne_formatting_sample_title_authors_affiliations.pdf*](https://journals.plos.org/plosone/s/file?id=ba62/PLOSOne_formatting_sample_title_authors_affiliations.pdf)*.*

Thank you for the clarification and direction on the *PLOS ONE* style requirements. On further review of the provided title page template, we have removed the “Full Title” and “Short Title” sections on lines 1-6 (1-2). We have also added clarification on equal contributions for each author on line 9 (5,16-17).

We have changed our figure and supplemental figure file names to better comply with the desired file naming conventions as outlined in the *PLOS ONE* guidelines. Each figure we have changed from “Figure 1” to “Fig1”, and the supplemental figures and tables have been changed to include the S numbering system (e.g. S1, S2, etc.).

We have changed the main sections to a 14pt bolded font, and each subsection to a 11pt bolded font instead of using the numbering convention. If this is not the desired alteration, we can also change it back based upon your feedback.

*2. To comply with PLOS ONE submissions requirements, in your Methods section, please provide additional information regarding the experiments involving animals and ensure you have included details on methods of anesthesia and/or analgesia.*

We appreciate the feedback on this aspect of our methods section. To address this concern, we have included our handling practices, and chute brand/design for animal restraint on lines 129-130 (123-124). Additionally, we have added verbiage in the Study Population section about our use of xylazine and efforts to reduce animal suffering on lines 119-121 (113-115). Specific doses and brands used can be found on lines 137-140 (131-134). Based on our interpretation of *PLOS ONE* ‘s Animal Research guidelines, we have made every effort to include desired information regarding sedation and analgesia, but if there is additional information that is desired, we can supply it as needed.

*3. Thank you for uploading your study's underlying data set. Unfortunately, the repository you have noted in your Data Availability statement does not qualify as an acceptable data repository according to PLOS's standards.*

*At this time, please upload the minimal data set necessary to replicate your study's findings to a stable, public repository (such as figshare or Dryad) and provide us with the relevant URLs, DOIs, or accession numbers that may be used to access these data. For a list of recommended repositories and additional information on PLOS standards for data deposition, please see* [*https://journals.plos.org/plosone/s/recommended-repositories*](https://journals.plos.org/plosone/s/recommended-repositories)*.*

Thank you for this clarification on acceptable repositories. We have elected to upload our dataset to the Dryad repository and still include the GEO accession number so that readers can access both at their discretion. The Dryad repository reviewer link has been shared elsewhere in the submission, and can also be found here: [http://datadryad.org/share/LINK_NOT_FOR_PUBLICATION/tFIZElhw0_WoF02Q1mmUKXPf3N9FbTHBVUYc6RGNbNw](https://nam10.safelinks.protection.outlook.com/?url=http%3A%2F%2Fh52wvrds.r.us-west-2.awstrack.me%2FL0%2Fhttp%3A%252F%252Fdatadryad.org%252Fshare%252FLINK_NOT_FOR_PUBLICATION%252FtFIZElhw0_WoF02Q1mmUKXPf3N9FbTHBVUYc6RGNbNw%2F1%2F010101998cda146b-44014d39-74db-4af4-a060-a0cc6faabd64-000000%2F5YwafV6uM0Eju8J89Kx1dTEQvZg%3D446&data=05%7C02%7Cgrace.jakes%40colostate.edu%7C4dbcdc0b91524271bd5b08ddfe03ff46%7Cafb58802ff7a4bb1ab21367ff2ecfc8b%7C0%7C0%7C638946015318072074%7CUnknown%7CTWFpbGZsb3d8eyJFbXB0eU1hcGkiOnRydWUsIlYiOiIwLjAuMDAwMCIsIlAiOiJXaW4zMiIsIkFOIjoiTWFpbCIsIldUIjoyfQ%3D%3D%7C0%7C%7C%7C&sdata=J9XylMYTi%2BKkrW6WVyNmhDlBCNTsxUMulZy9qW%2FktNg%3D&reserved=0).

The DOI link for publishing has been included in the body of the manuscript on line 225 (217), and can also be found here:

<https://doi.org/10.5061/dryad.02v6wwqh6>.

*4. Please upload a copy of Supporting Information Table 2 which you refer to in your text on page 23.*

We apologize for this oversite and appreciate you bringing this to our attention. We have uploaded this table, now as Supporting Information/Table 4, as we have added more supporting information earlier in the document.

*5. If the reviewer comments include a recommendation to cite specific previously published works, please review and evaluate these publications to determine whether they are relevant and should be cited. There is no requirement to cite these works unless the editor has indicated otherwise.*

We did not see any reviewer comments pertaining to citing other published work, but if further in the review process there is a request for other citations, we would be more than happy to address these as they arise.

**Response to Reviewer 1:**

Thank you for the time and effort it took to review our manuscript. Your thoughts and comments were extremely helpful in the revision of our manuscript, and we have made every effort to address each point raised in the review process. In our response to your comments, we have included your prior comment in italics, and our response in normal font immediately following that paragraph. We have sought to thoughtfully address every point raised and can provide more support or clarification to our data and revisions as needed.

1) *The core finding that stress induces, rather than suppresses, inflammation differs somewhat from the understanding of BRD, but I think that idea is not totally novel and is consistent with prior studies in calves (e.g., Hodgson et al., Griebel’s work), many of which the authors identified. This manuscript extends that work by focusing specifically on the lower airway and linking inflammation to pathogen-independent mechanisms, demonstrated by the absence of detectable pathogens in BALF at the time of sampling. The authors should consider softening the novelty claims around increased vs. suppressed inflammation, and instead emphasize the value of this study in refining prior observations through a mucosal, pathogen-exclusion lens.*

We appreciate the perspective in this comment and agree that our initial language may have over accentuated the novelty of our data, specifically in the context of stress. In response to this comment, we have altered our language in the abstract, introduction, and discussion sections of the manuscript to soften and redirect our commentary to focus on the mucosal host-pathogen aspect of our findings. Please see our changes at lines 43-44 (38-40), 94 (88-89), 399-409 (382-392).

2) *The authors heavily emphasize the CIBERSort cell deconvolution data in the discussion. However, this data and analysis is not clearly described and lacks validation. More detail is needed on the reference dataset used, and the markers used to differentiate these populations. Without supporting cytology, flow cytometry, or BALF cell counts, the inferred shift in neutrophils should be interpreted cautiously and some discussion should be added regarding the limitations of relying solely on computational predictions should be acknowledged. Along these lines, Figure 7 doesn't seem to add much to the discussion section.*

Thank you for this comment. We have added documentation detailing our methods and markers used in this CIBERSORTx analysis. Specifically, Table 1 in the body of the document has been added to detail select markers used to classify cellular populations. This does not provide an exhaustive list of the classifications, rather is intended to be similar to a flow cytometry-style panel, demonstrating upregulated and “negative” or downregulated markers. We have used both cell surface markers and transcription factors to classify the cellular populations and have included a minimum of 3 upregulated markers per cell type. A full list of the defining upregulated and downregulated markers for each cell type can be found in Supplemental Table 1. We have also visually represented the dataset using a uniform manifold approximation and projection (UMAP) image so that the readers can see the distribution and make-up of the single-cell dataset (Supplemental Figure 1a and 1b). A dot plot heat map has been included as a visual representation of the markers used for each cell type (Supplemental Figure 1c). The numbers/columns on the dot plot represent the individual cell clusters on the UMAP, so that readers may cross-reference the distribution of cell populations in relation to one another and their major markers.

Several of the calves included in the study had paired single-cell and bulk sequencing samples, so we completed a difference from single-cell “ground truth” analysis on our CIBERSORTx predicted values for each cell type. We completed this analysis for both of the different batch correction methods evaluated and have included that data in Supplemental Figure 2. We also conducted a correlation analysis and have included the visual representation of this analysis as well as the R^2^ values in Supplemental Figure 3.

A detailed discussion of our CIBERSORTx methodology can be found in the Methods section, lines 197-222 (189-214). Please let us know if additional support or clarification of the data is needed.

We have added a paragraph discussing the limitations of *in silico* predictions of cell population proportions on lines 347-351 (336-340) in the Discussion section, and upon further reflection, have elected to remove primary Figure 7 from the manuscript.

**Response to Reviewer 2:**

Thank you for the time and effort it took to review our manuscript. Your thoughts and comments were extremely helpful in the revision of our manuscript, and we have made every effort to address each point raised in the review process. In our response to your comments, we have included your prior comment in italics, and our response in normal font immediately following that paragraph. We have sought to thoughtfully address every point raised and can provide more support or clarification to our data and revisions as needed.

*Introduction Line 83-91: There is no mention of the fact that haptoglobin, cortisol, LBP, and SAA would be measured in the blood of study cattle. What objective was being addressed by these measurements? That should be added to this section.*

We appreciate this feedback. We sought to use these markers to demonstrate that calves in this study were truly stressed, or at least responding systemically to the transit and auction stressors that they had experienced. As we were not in control of exactly when the calves would arrive and be sampled (beyond within the first 18-24 hours after arrival), we chose multiple stress markers that covered early and later stress responses. We have updated the manuscript to reflect this in lines 87-88 (82-83).

*Materials and Methods Where are the methods for the CIBERSORT? They need to be included.*

Thank you for this clarifying request. We have included detailed methodology on lines 197-222 (189-214). We also wanted to respond with some explanation for each supplemental figure and table added for the CIBERSORTx methods:

Table 1 in the body of the document has been added to detail select markers used to classify cellular populations. This does not provide an exhaustive list of the classifications, rather is intended to be similar to a flow cytometry-style panel, demonstrating upregulated and “negative” or downregulated markers. We have used both cell surface markers and transcription factors to classify the cellular populations and have included a minimum of 3 upregulated markers per cell type. A full list of the defining upregulated and downregulated markers for each cell type can be found in Supplemental Table 1. We have also visually represented the dataset using a uniform manifold approximation and projection (UMAP) image so that the readers can see the distribution and make-up of the single-cell dataset (Supplemental Figure 1a and 1b). A dot plot heat map has been included as a visual representation of the markers used for each cell type (Supplemental Figure 1c). The numbers/columns on the dot plot represent the individual cell clusters on the UMAP, so that readers may cross-reference the distribution of cell populations in relation to one another and their major markers.

Several of the calves included in the study had paired single-cell and bulk sequencing samples, so we completed a difference from single-cell “ground truth” analysis on our CIBERSORTx predicted values for each cell type. We completed this analysis for both of the different batch correction methods evaluated and have included that data in Supplemental Figure 2. We also conducted a correlation analysis and have included the visual representation of this analysis as well as the R^2^ values in Supplemental Figure 3.

*Were the calves in the Stressed group and the calves in the Acclimated group all from the same group of cattle purchased at the same time? Or were they purchased at different times?*

Thank you for this question. We randomly sampled cattle in batches of 3-4 from different shipments throughout the purchasing season. This was done to ideally get the most randomized and representative sample from the backgrounding period, not biased by calf source. We have updated the manuscript to reflect this on line 103-104 (97-98).

*Did the cattle receive antimicrobial metaphylaxis (i.e. long acting antimicrobial at arrival for BRD control)? This should be explicitly stated.*

Thank you for this question. The calves did not receive any metaphylaxis, nor did any calf that arrived or was put out to pasture at the same time as the enrolled calves. We have added this information on line 112 (106).

*The study design is not very clear. Is it true that some Stressed calves were never diagnosed with BRD after they were sampled until the end of the backgrounding period, while others were diagnosed with BRD (and sampled when they were)?And is it true that the Acclimated calves were never diagnosed with BRD between arrival and the day they were sampled? If so, it would help improve reader understanding of the design if this was stated explicitly. Also, how long was the total backgrounding period?*

We appreciate this feedback and apologize for the lack of clarity. Yes, 7 of the Stressed calves were never diagnosed with BRD throughout the backgrounding period. Calves that developed BRD within 14 days of arrival were sampled at the time they were pulled (before antibiotic administration). Two calves developed BRD ~6 weeks after arrival and were not sampled because the backgrounder could not coordinate with our sampling over the Thanksgiving holiday. We have updated the manuscript to address this briefly on lines 158-160 (151-152).

Acclimated calves were never diagnosed with BRD between arrival and when they were sampled (and through the entire backgrounding period). We have updated the manuscript to reflect this on line 149-152 (142-145).

*Reader understanding of the experimental design could be improved by adding more detail about the experimental design to Figure 1. For example, did all Stressed steers subsequently develop BRD, and have BAL collected when they developed BRD, or only some of them? For each Stressed animal sampled that developed BRD, how many days after arrival did BRD occur?*

Thank you for this suggestion. We have substantially changed Figure 1 to include the timeline of sampling/the backgrounding period, and to include the number of Stressed calves who developed BRD and were sampled again. We included the date range for when we sampled calves who had developed BRD, and the specific number of days can be found in Supplemental Table 2.

*Line 98-100: power analysis described, but what outcome was the focus of the power calculation, and how much difference between the 2 groups in this outcome would the sample size of 9 detect?*

We appreciate this clarifying question. We based our power analysis on expected differences in acute-phase proteins and cortisol, as these were our validation markers for stress. We derived our expected difference between the two groups from recently published literature (Beenken et al., 2021 in our references, among others) and anticipated that each of the stress mediators would be elevated in the study animals. We were incorrect about cortisol and LBP, but we addressed theories on why this could be (time of sampling, possible lack of LPS stimulation) in the Discussion. The details regarding specific values for the difference we were expecting to see can be found on lines 103-109 (98-104).

*Line 123: "foley" should be capitalized, and it seems that the term "Foley catheter" is limited to the type of cuffed catheters used for urinary catheterization. It may be more correct to simply to say "A sterile cuffed bronchoalveolar lavage catheter..." instead of "..Foley cather..."*

Thank you for this correction. We have updated our manuscript at line 140 (134) to reflect your recommended change.

*Lines 182-186: what statistical test was used to compare cortisol and acute phase proteins between groups?*

We used the Mann-Whitney U test and have updated the manuscript at line 235 (227) to reflect this.

*Given the small number of cattle included in this study, the authors should provide metadata for each animal in both the Stressed and Acclimated groups in a spreadsheet provided as supplementary data. The metadata should information such as the weight of each animal, the day relative to arrival when the BAL and blood were collected, and all times the animal was treated for BRD or any other disease, and what treatment they received (i.e. which antimicrobial)*

Thank you for this recommendation. We have included this metadata in Supplemental Table 2, with the exception of animal weight, as that was not recorded by the backgrounder, and we were not able to collect that data at the time of sampling.

*Where is the list of all DEG? They should be provided in a spreadsheet as supplementary data.*

The complete list of DEGs can be found in Supplemental Table 3. It is sorted in order of Log2 Fold Change, and includes the p value of each gene, as well as the FDR (padj column).

*Line 227: "...whether they developed BRD in the first week after arrival..." Do the authors mean "first 2 weeks", as indicated in Supplementary Figure 4?*

Thank you for bringing this to our attention. Yes, this was a typographical error that we have corrected on line 258-259 (250).

*Line 255: "Clade" refers to a group of organisms that appear to have evolved from the same ancestor; it does not appear to be an appropriate term as used here. The word "clade" should be replaced with a different word in the manuscript and figures; "group" is probably adequate.*

Thank you for this note of correction. We have changed the word “clade” to “group” as recommended, and the change can be found on line 289 and line 294 (281).

*Figure 1: As mentioned above, Figure 1 could be made more informative by inclusion of more information, e.g., how many cattle in each group was treated for BRD and when. It is not clear that as currently presented Figure 1 provides enough information to warrant inclusion.*

We very much appreciate this feedback. We have altered the figure to reflect the sampling timeline through the backgrounding period, to include indications of when and how many Stressed animals were sampled post BRD diagnosis. Please let us know if we can add more clarifying features to this graphical representation of our study design.

*Figure 5: Some of the text of the legend of Figure 5 is unclear, what does this sentence mean: "One Stressed calf displaying the most profound inflammatory signaling was removed for this representation to demonstrate the clades of inflammatory signaling"? Please revise this legend to make it more clear. It is not very clear why the heatmap in Figure 5 is presented in the body of manuscript and the heatmap in Figure 4 was presented as supplementary data. Why not just present the heatmap in Figure 4 in the body of the manuscript?*

Thank you for this comment. Per this feedback, we have elected to switch Supplemental Figure 4 (now S7) with primary Figure 5. Initially we felt that the heat map representation, including the calf with the greatest inflammatory signaling, diminished the visual representation of how calves separated into groups based upon levels of inflammatory signaling. We still feel that seeing the groups better is aided by inclusion of Supplemental Figure 7, but we agree that it is more appropriate to include the representation with all calves in the body of the manuscript.

*The asterisks in Supplementary Fig 4 indicates cattle treated for BRD within 14 days of arrival; were any treated after 14 days but before the end of the backgrounding period?*

Yes, there were two calves that were treated approximately six weeks after arrival at the facility. We elected to not to analyze these calves as “BRD” calves in our DEG analysis found in Supplemental Figure 6 because we felt that it was less likely that stress was principally affecting their immune milieu at the point of BRD diagnosis. Our goal with the DEG analysis was to find immune predictors of BRD development in the first 2-3 weeks at a new facility, as BRD diagnosis after those first weeks would likely be further complicated by other factors including grazing and weather dynamics, etc. Further, due to technical constraints around the Thanksgiving holiday, we were not able to sample these calves to determine the etiology of BRD signs. Therefore, we felt that since we could not speak to the etiology or contributing factors of BRD development, we would limit our downstream analysis to classifying calves based upon the development of signs within the first 2-3 weeks at the facility.

*Figure 7: In the image of the alveolus of the stressed animal, what do the small green dots represent?Because the mechanism depicted in Figure 7 is entirely hypothetical, it is not clear that this figure is truly warranted.*

The green dots were intended to represent cytokine release/production, however since both reviewers indicated that Figure 7 may not be warranted, we elected to remove this figure from the revised manuscript.

*Supplemental Figure 3 has two components, A and B, but these are not both described in the legend for this figure.*

Thank you for this comment. We have added clarifying verbiage in the caption for this figure (now Supplemental Figure 6) on lines 570-571 (550-551).

**Concluding Remarks**

We want to reiterate our appreciation for the consideration of our manuscript for publication in *PLOS ONE*, and the time and effort it took you and the reviewers to provide constructive feedback. We have sought to address each point raised in the review process to the best of our ability and will continue to do so through the rest of the review process. Please let us know if there are other data needs or ways that we can clarify the manuscript as it stands now.

Thank you again for your consideration,

Grace Jakes (1^st^ author)

Sarah Raabis (co-corresponding author)
