# Supplementary material for: Transport stress induces paradoxical increases in airway inflammatory responses in beef stocker cattle
Source: PLoS One. 2026 Feb 6;21(2):e0328428. doi: 10.1371/journal.pone.0328428 (PMC12880702; doi:10.1371/journal.pone.0328428)
Supplement: Attachment — Submitted filename: Response_to_Reviewers_auresp_2.docx [file pone.0328428.s013.docx]

Dear Dr. Abuelo,

Thank you for your continued consideration of our manuscript entitled, “Transport stress induced paradoxical increases in airway inflammatory responses in beef stocker cattle.” We sincerely appreciate the feedback included in this request for edits. We have sought to address these points individually and to the best of our ability and will appreciate any additional feedback should it arise.

Similar to our first round of edits, any in-text changes will be cited by line number in the “Revised Manuscript with Track Changes” document, and then in parenthesis for the Manuscript document (e.g. lines 115-117 [105-107]). We are only aware of one request for an in-text edit, as requested by Reviewer 2. We have made a few minor edits to our reference list based upon closer review of the style guidelines and have included specifics on those edits in the Response to Editorial comments section. Included in the editorial comments was a request to ensure that no retracted articles were included in our list of references. We are unaware of any retracted articles included in our list of references but would make every effort to remove and replace such a reference should they exist.

Please see our specific responses to editorial and reviewer comments in the following sections. Should any response not satisfactorily address a point raised by the editors or reviewers, we would be more than happy to revisit any point more thoroughly if indicated.

**Response to Editorial Comments:**

1. *If the reviewer comments include a recommendation to cite specific previously published works, please review and evaluate these publications to determine whether they are relevant and should be cited. There is no requirement to cite these works unless the editor has indicated otherwise. Please review your reference list to ensure that it is complete and correct. If you have cited papers that have been retracted, please include the rationale for doing so in the manuscript text, or remove these references and replace them with relevant current references. Any changes to the reference list should be mentioned in the rebuttal letter that accompanies your revised manuscript. If you need to cite a retracted article, indicate the article’s retracted status in the References list and also include a citation and full reference for the retraction notice.*

Thank you for this opportunity to review our submitted references. Upon further review, we have edited the journal names to include the NLM title abbreviation rather than the full journal name for each entry. We apologize for our incorrect interpretation of the style guidelines in our initial submission. We have also slightly edited the USDA report entries to better meet the citation guidelines. Finally, the pwr package citation has been edited to link to the CRAN repository rather than the author’s GitHub page, as based upon further review we believe that this may align more correctly with Vancouver citation guidelines. There was one citation (Nyhlén et al., 2000) where the citation was inadvertently included in caps rather than sentence case, and that has been corrected. We regret this oversite.

We have made every effort to review our list of references to make sure that no retracted articles are included in our reference list. We are unaware of any article(s) that have been retracted and would be more than willing to remove any reference that is retracted should that be the case.

We have not received any requests from reviewers to cite additional work, but if there are gaps in our discussion or introduction that should be filled, we would be more than happy to review any relevant literature not already included if indicated.

**Response to Reviewer 1:**

Thank you for your careful review of our revised manuscript. We appreciate your effort in the review process and would welcome any further comments should they arise.

**Response to Reviewer 2:**

Thank you for your careful consideration of this revised manuscript draft. We appreciate your comments and have done our best to address each one.

Thank you for catching the mislabeling/reference to Supplemental Figure 5. We have corrected this in line 245 (245) of the Manuscript.

We also appreciate your suggestion of including the ultrasound score and rectal temperature at the second BAL sampling, when calves were diagnosed with BRD by pen-riders, in our supplemental data. This has been included in Supplemental Table 2 (S2 Table) under columns “Rectal Temperature at Second BAL (degrees C)” and “Lung Ultrasound Score at Second BAL.” It is important to note that the ultrasound score was 0 on two of the three calves, but based upon rectal temperature, behavior, upper respiratory signs, and caretaker best-judgement, these calves were diagnosed with BRD and were treated. The identification and treatment of these calves prior to the development of severe lung lesions is likely a testament to their acute development of signs and timely identification by pen riders before severe pulmonary disease could develop. Additionally, ultrasound examination was limited in this study, as we were unable to visualize the right cranial lung lobe consistently due to the logistics of the chute design (see lines 129-130). Therefore, lesions may have been present and not detected by ultrasound exam.

We want to reiterate our appreciation of your time and effort in reviewing our manuscript. Should you identify any further changes in the review process, we would be more than happy to address them as they arise.

**Concluding Remarks**

We want to conclude by reiterating our appreciation for the editorial and reviewer comments. They have substantially advanced the quality of our submission.

Thank you again for your consideration,

Grace Jakes (1^st^ author)

Sarah Raabis (co-corresponding author)
